# The Tumour Vasculature as a Target to Modulate Leucocyte Trafficking

**DOI:** 10.3390/cancers13071724

**Published:** 2021-04-06

**Authors:** Yang Zhao, Ka Ka Ting, Paul Coleman, Yanfei Qi, Jinbiao Chen, Mathew Vadas, Jennifer Gamble

**Affiliations:** 1 Department of Biochemistry and Molecular Biology, School of Medicine & Holistic Integrative Medicine, Nanjing University of Chinese Medicine, Nanjing 210023, China; 2 Vascular Biology Program, Centenary Institute, The University of Sydney, Sydney 2050, Australia; k.ting@centenary.org.au (K.K.T.); p.coleman@centenary.org.au (P.C.); j.qi@centenary.org.au (Y.Q.); m.vadas@centenary.org.au (M.V.); 3 Liver Injury and Cancer Program, Centenary Institute, The University of Sydney, Sydney 2050, Australia; j.chen@centenary.org.au

**Keywords:** tumour vasculature, leucocyte trafficking, vascular normalisation, immunotherapy

## Abstract

**Simple Summary:**

Tumour blood vessels, characterised by abnormal morphology and function, create an immunosuppressive tumour microenvironment via restricting the appropriate leucocyte subsets trafficking. Strategies to trigger phenotypic alteration in tumour vascular system to resemble normal vascular system, named vascular normalisation, promote effective trafficking of leucocytes into tumours through enhancing the interactions between leucocytes and endothelial cells. This review specifically demonstrates how targeting tumour blood vessels modulates the critical steps of leucocyte trafficking. Furthermore, selective regulation of leucocyte subsets trafficking in tumours can be achieved by vasculature-targeting strategies, contributing to improved immunotherapy and thereby delayed tumour progression.

**Abstract:**

The effectiveness of immunotherapy against solid tumours is dependent on the appropriate leucocyte subsets trafficking and accumulating in the tumour microenvironment (TME) with recruitment occurring at the endothelium. Such recruitment involves interactions between the leucocytes and the endothelial cells (ECs) of the vessel and occurs through a series of steps including leucocyte capture, their rolling, adhesion, and intraluminal crawling, and finally leucocyte transendothelial migration across the endothelium. The tumour vasculature can curb the trafficking of leucocytes through influencing each step of the leucocyte recruitment process, ultimately producing an immunoresistant microenvironment. Modulation of the tumour vasculature by strategies such as vascular normalisation have proven to be efficient in facilitating leucocyte trafficking into tumours and enhancing immunotherapy. In this review, we discuss the underlying mechanisms of abnormal tumour vasculature and its impact on leucocyte trafficking, and potential strategies for overcoming the tumour vascular abnormalities to boost immunotherapy via increasing leucocyte recruitment.

## 1. Introduction

The presence of specific subtypes of leucocytes in tumours is closely related to improved prognosis for cancer patients [1]. The ability of leucocytes to perform immune surveillance relies on their potential to infiltrate into the tumour, and penetrate into the tumour parenchyma [2]. The tumour endothelium is recognised as a hub for controlling the trafficking of leucocytes into solid cancers. As for a normal inflammatory or immune response, leucocyte trafficking occurs through a multiple cascade of events starting with leucocyte capture onto the tumour endothelial cells (ECs), their rolling and firm adhesion on the activated endothelium, intraluminal crawling of the leucocytes, and finally their transmigration across the endothelial barrier. This transmigration can occur via either the paracellular route generated between two adjacent ECs, or the transcellular route, through the EC cell body [3,4]. Targeting tumour endothelium to enhance selective recruitment of leucocyte subsets to convert a ‘cold’ tumour to a ‘hot’ tumour may represent an effective avenue to improve immunotherapy and hence combat tumour progression.

The imbalance of angiogenic mediators in tumours is prone to strengthen tumour angiogenesis and elicit the tumour blood vessels to develop abnormally, in terms of structure and function. The aberrant tumour vasculature plays an important role in generating an immune suppressive microenvironment, preventing the trafficking of leucocytes from infiltrating into the tumour bed. Anti-angiogenesis therapy can result in altered leucocyte trafficking to enhance immunotherapy. However, the overall clinical achievement of anti-angiogenic agents has been less than what was expected. This lack of benefit for cancer patients appears to be due to “intrinsic resistance” (resistance to anti-angiogenic therapies observed at the beginning of the treatment) and “acquired resistance” (produced resistance after an initial response to anti-angiogenic therapies) to the drugs [5]. In recent years, vascular normalisation, defined as reversing abnormal tumour blood vessels back to normal blood vessels, has gained increasing attention due to its potent efficiency and limited side effects. It can restore the angiogenic vessels towards a mature and stable vasculature with improved vascular perfusion and reduced vascular permeability, offering an appropriate microenvironment for promoting the trafficking of leucocytes [6]. Notably, normalised tumour blood vessels can selectively control the infiltration of leucocyte subpopulations, enhancing the anti-tumour immunity [7].

In this review, we summarise the current knowledge on the role of tumour blood vessels in controlling leucocyte trafficking, importantly on the selective trafficking of subpopulations of leucocytes. We discuss potential therapeutic strategies for modulating tumour vasculature to influence leucocyte recruitment.

## 2. The Tumour Vasculature: An Important Mediator of a Suppressive Tumour Microenvironment

### 2.1. The Structurally and Functionally Aberrant Tumour Vasculature

Normal blood vessels are composed of evenly spaced and well-differentiated arterioles, capillaries, and venules [8]. In contrast, the tumour vasculature exhibits a chaotic pattern of disorganisation, mainly due to the uncontrolled growth of the neoplastic cell population and associated overexpression of pro-angiogenic factors [9,10]. Tumour blood vessels are dilated, tortuous and heterogeneous. They possess irregular branches with uneven diameters and frequently form arterio-venous shunts. The structure of the vessel wall also displays abnormalities with wide inter-endothelial junctions and elevated numbers of fenestrations [11]. Tumour blood vessels also have less pericyte coverage as a result of reduced pericyte recruitment and these are loosely connected to the ECs [12]. Similarly, the basement membrane support of blood vessels in tumours is conspicuously abnormal, either irregularly thick or totally absent [13].

The abnormalities in tumour vascular structure contribute to an immature and dysfunctional tumour vasculature. Increased expression of angiogenic factors, in particular vascular endothelial growth factor (VEGF) results in disrupted junctions and as such, tumour blood vessels are extremely permeable to intravascular fluids with resulting increased interstitial fluid pressure (IFP) [14]. In addition, vascular perfusion in tumours is compromised and unevenly distributed, caused by vessel tortuosity and compression. The presence of poor perfused regions in tumours gives rise to a hypoxic and low pH tumour microenvironment (TME), which impedes the delivery of chemotherapeutic drugs and efficacy of immunotherapy [15,16].

### 2.2. The Immunoresistant Microenvironment

A growing body of evidence has revealed that unproductive and aberrant tumour-associated blood vessels emerge as an important contributor to the tumour immunoresistant microenvironment. More precisely, tumour blood vessels not only influence how cancer cells escape the anticancer immunosurveillance and respond to immunotherapy, but they also determine the number and activity of leucocytes that invade into tumours [17,18,19]. Structural and functional abnormalities in tumours confer an uneven blood flow and pressure, restricting the effective penetration of tumour infiltrating lymphocytes (TILs) [16,20]. Additionally, a hypoxic and acidotic microenvironment caused by the aberrant tumour vasculature decreases the infiltration of immune-supportive cell populations (e.g., T lymphocytes, natural killer cells, and dendritic cells), but facilitate attraction of immunosuppressive immune cells (myeloid-derived suppressor cells, neutrophils, etc.) [15,21]. Of note, tumour ECs are characterised by a pro-angiogenic phenotype with the upregulation of a range of angiogenesis-related tyrosine kinase receptors like VEGF receptor 1 (VEGFR1), VEGF receptor 2 (VEGFR2), platelet-derived endothelial growth factor receptor (PDGFR) and endothelial growth factor receptor (EGFR), which specifically inhibit tumour immunity [22,23,24]. The microenvironment also results in downregulation of intercellular adhesion molecule 1 (ICAM1) and vascular cell adhesion molecule 1 (VCAM1), adhesion molecules required for immune effector cell extravasation [2]. The functional activity of the TILs is also changed by the tumour ECs. Tumour ECs are able to selectively upregulate inhibitory receptors of T cell activation including programmed cell death protein ligand 1 (PD-L1), programmed cell death protein ligand 2 (PD-L2), T-cell immunoglobulin mucin-3 (TIM3), and B7-H3 (also known as CD276) [25,26,27]. Furthermore, tumour necrosis factor-related apoptosis inducing ligand (TRAIL) and Fas ligand (FasL) can be expressed on tumour ECs, selectively killing effector T cells [28]. Abnormal tumour vasculature is also believed to impede the delivery of chemotherapeutics and immunotherapeutic entities [14]. Taken together, tumour blood vessels play an inhibitory role in the penetration of effector immune cells into tumour parenchyma and create an immunoresistant microenvironment.

### 2.3. Vascular Normalisation

Vascular normalisation has been regarded as an effective vascular-targeting therapeutic strategy since it was first described by Rakesh Jain and colleagues [6,14]. Instead of pruning tumour blood vessels as anti-angiogenesis achieves, vascular normalisation reverses the abnormal tumour vascular system back to a more normal vascular system, with restoration of structural and functional vascular integrity [16,29]. Indeed, numerous agents have been reported to fulfill tumour vascular normalisation in preclinical and clinical studies. These include bevacizumab, vanucizumab, angiopoetin-2 binding, and Tie2 activating antibody (ABTAA) and NGR-TNFα [30,31,32,33].

**Figure 1:**
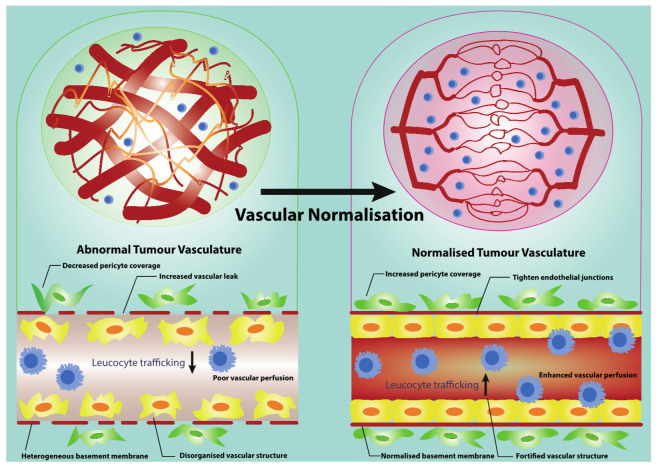
Vascular normalisation serves as an effective vasculature-targeting therapy. Vascular normalisation transforms angiogenic blood vessels towards a stabilised vasculature. The stabilised tumour vasculature shows elevated pericyte coverage and normal basement membrane support. The fortified vascular structure also leads to the reduced vascular permeability, increased vascular perfusion and ameliorated hypoxia. All of these give rise to enhanced leucocyte trafficking.

In the process of vascular normalisation, disorganised and highly proliferating tumour ECs become more quiescent and less active. The normalised endothelium tends to form tighter interendothelial junctions between neighbouring cells involving adherens junction molecules such as vascular endothelial (VE)-cadherin and tight junction (e.g., zonula occludens (ZO)-1, ZO-2, and Claudin-5) [34]. Pericyte coverage is dramatically enhanced and basement membrane appears to be more normalised, leading to the fortified and mature vascular network. Importantly, tumour vascular function is improved, as reflected by increased vascular perfusion, decreased vascular permeability, and alleviated hypoxic microenvironment (Figure 1) [16,35]. The normalised tumour vasculature generates an immune-supportive microenvironment through selectively modulating the infiltration of immune-associated cell populations into tumours. In particular, vascular normalisation promotes the infiltration of T lymphocytes, dendritic cells (DCs), and natural killer (NK) cells while impeding the penetration of neutrophils, regulatory T cells (Tregs) and myeloid derived suppressor cells (MDSCs) [7]. It is also able to enhance the immune response via improving the function of immune cells such as skewing tumour-associated macrophage (TAM) polarisation from a M2 phenotype toward a tumour-inhibiting M1 phenotype [36,37]. Thus, normalised ECs appear to efficiently respond to pro-inflammatory signalling and express sufficient levels of molecules that are involved in the leucocyte capture, adhesion, and extravasation process [38].

Vascular normalisation, however, appears to be dose-dependent. For example, high doses of anti-angiogenic drugs (such as DC101, Ramucirumab, an anti-VEGF Receptor 2 antibody) are prone to destroy the tumour blood vessels with impaired drug delivery whilst too low a dose fails to work on tumour blood vessels [18,39]. Further, vascular normalisation exhibits a transient effect, as prolonged inhibition of angiogenesis leads to substantial pruning of the vascular system. Excessive pruning of tumour blood vessels may be detrimental as a result of increased hypoxia [40]. Hence, vascular normalisation requires judicious dosing and an appropriate timeframe to render a “normalisation window” within which vascular perfusion is improved and immunotherapy is enhanced [18]. However, the window for normalising the tumour vasculature is generally short and not easily identifiable, and often variable between patients. Hence, markers representing the effective therapeutic window for vascular normalisation are needed [41].

## 3. Leucocyte Trafficking 

**Figure 2:**
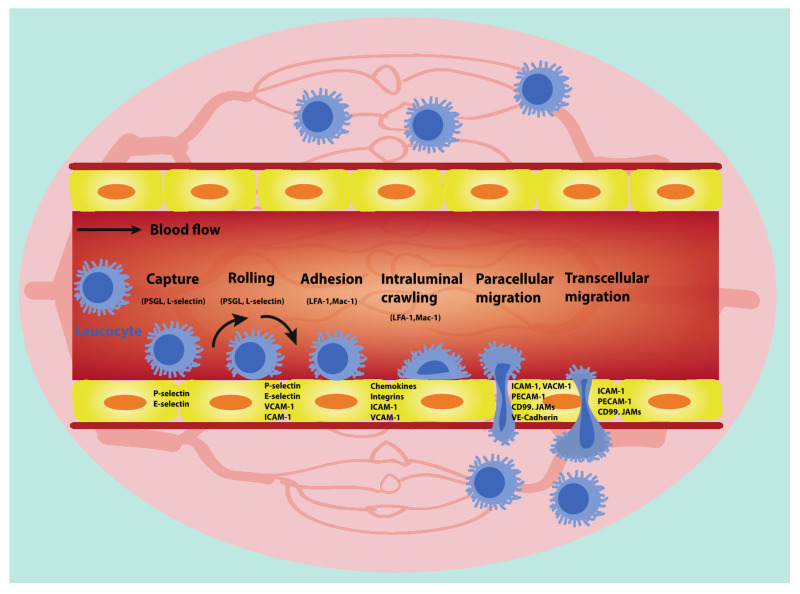
The process of leucocyte trafficking. The trafficking of leucocytes involves a cascade of indispensable events including leucocyte capture, rolling, adhesion, intraluminal crawling and transmigration (paracellular migration and transcellular migration). All of these steps require the interactions between leucocytes and endothelial cells (ECs). A selected set of molecules involved in the leucocytes–ECs interaction cascade are presented.

Leucocytes use an array of cell adhesion molecules and chemokines to attach and transmigrate across ECs in order to penetrate into connective tissue stroma at an inflammatory site (Figure 2) [3]. The process proceeds from tethering (capture), followed by rolling, adhesion, intraluminal crawling and is completed by paracellular or transcellular migration across the endothelium [42]. The transient interaction between leucocytes and ECs as well as the impact of blood flow elicits the rolling of leucocytes on the apical EC surface, giving rise to pulling of long membrane tethers at the rear of the rolling leucocytes. Chemokines and other chemoattractants expressed on the EC surface are able to induce the activation of rolling leucocytes. The EC–leucocyte interaction induced by critical adhesion molecules governs firm adhesion and further crawling of the leucocytes until they transmigrate across the endothelial barrier [43,44].

Tumours demonstrate specific tumour microenvironments characterised by differential chemokines and chemotactic factors that affect leucocyte recruitment [45]. Leucocytes migrate in a directed manner along the concentration gradient of the chemokine or chemoattractant towards the inflammatory site. In tumours, these processes are aberrant, resulting in either reduced or skewed leucocyte subset accumulation into the tumour parenchyma [1,18,46,47,48]. Nonetheless, tumours also appear to be diverse regarding the expression of molecules involved in leucocyte trafficking (homing-associated molecules). Some tumours present quite an inflammatory type of endothelium with high levels of adhesion molecules expression, and this has been deemed to have a prognostic impact [49].

### 3.1. Leucocyte Capture

In a normal inflammatory response, the initial capture of leucocytes from the blood circulation is regarded as a prerequisite for leucocyte rolling along the adhesive endothelial surface [50] and can be considered as a rate-limiting step in leucocyte trafficking and accumulation. Leucocyte capture occurs through two routes: primary capture and secondary capture [51]. Primary capture refers to the process that leucocytes attach directly to the activated endothelium and then initiate rolling interactions. On the other hand, a freely flowing leucocyte can also transiently interact with a rolling leucocyte via an L-selectin-dependent mechanism and subsequently attach to the endothelium, which can be defined as secondary capture [50]. The initial contact of leucocytes with ECs appears to be strictly physical in nature. For instance, shear thresholding precludes secondary capture at low shear stresses while amplifying it at high shear stresses [52]. Further, the occurrence of capture is strongly dependent on the molecular interactions between the ECs and leucocytes, in particular a series of critical mediators (e.g., P-selectin, E-selectin) in ECs play a pivotal role in capturing leucocytes via molecular bindings (Table 1).

An essential mechanism of tumour escape from immunity is the fact that tumour ECs fail to capture leucocytes from the blood circulation, thereby limiting the number of immune cells within the tumour mass [53,54,55]. Tumour ECs fail to adequately respond to many pro-inflammatory signaling stimulation, and fail to express sufficient levels of molecules involved in leucocyte capture, namely P-selectin and E-selectin. Moreover, physiological shear forces are essential for the trafficking of leucocytes. The architectural and structural defects of tumour vasculature prevent perfusion and also affect functional shear stress, thus dampening the capture of leucocytes [56,57]. Neutralising VEGF/VEGFR signalling in tumours is able to potentiate the efficacy and efficiency of cancer immunotherapy by elevating the expression of adhesion molecules and chemokines necessary for leucocyte capture [22,56,58].

### 3.2. Leucocyte Rolling and Adhesion

In general, the rolling of leucocytes on the luminal surface of ECs is predominantly governed by a cascade of carbohydrate binding proteins called E-, P-, and L-selectins, which are expressed on the surface of both ECs (E- and P-selectins) and leucocytes (L-selectin). E- and P-selectins can bind to glycosylated ligands on the leucocytes, most notably P-selectin glycoprotein ligand 1 (PSGL1) [59,60]. Both act to markedly diminish the velocity of leucocyte movement along ECs to allow for firm adhesion. Of note, the interplay between selectins and their ligands form and dissolve easily. Hence, selectins capture leucocytes, slowing their movement at the site of inflammation but still allow the leucocytes to roll over the surface of the endothelium under shear stress [61]. In fact, slow rolling seems to restrict the leucocytes into interactions with the ECs so that the leucocytes can be effectively activated by chemokines and other pro-inflammatory agents that are presented on the surface of the ECs [3]. Further interactions that lead to the slow rolling and firm adhesion are mediated through various endothelial adhesion molecules including VLA4, α4β7-integrin and mucosal vascular addressin cell adhesion molecule 1 (MAdCAM1), lymphocyte function-associated antigen 1 (LFA1), VCAM-1, and ICAM-1 (Table 2) [56].

The leucocyte–endothelial cell adhesion in tumour blood vessels is diminished under both basal and cytokine-stimulated circumstances [62]. Overproduction of pro-angiogenic factors such as VEGF in tumours can result in a decrease in the expression of adhesion molecules such as ICAM-1 and 2, VCAM-1, and CD34. This phenomenon is named “EC anergy” and results in the diminished leucocyte-vessel wall interactions, [54,63]. Tromp and colleagues demonstrated that a local release of either bFGF or VEGF impaired leucocyte adhesion in vivo through downregulation of endothelial adhesion molecules [64]. Furthermore, Bessa and co-workers showed that basal and lipopolysaccharide (LPS)-stimulated leucocyte rolling and adhesion were significantly decreased in tumour microvessels. They also found that ICAM-1 and transforming growth factor (TGF)-β1 immunoblockade partially and selectively reversed reduced leucocyte rolling and adhesion in tumour vasculature [65]. Of note, circulating leucocytes failed to appreciably interact with tumour microvessels without the stimulation of cytokines such as lymphotoxin. On the contrary, the number of fast and slow rolling leucocytes in tumour blood vessels was robustly increased in the presence of cytokine stimulation (this equally applies in normal vessels). Borgstrom et al. demonstrated that anti-P-selectin monoclonal antibody (mAb) treatment led to repression of fast rollers alone, whereas combination treatment with anti-P-selectin and anti-E-selectin mAbs efficiently prohibited slow rolling of leucocytes in tumour microvessels [66]. Additionally, it has been shown that nitric oxide (NO), a well-known physiological component of endothelial relaxation, could regulate blood flow perfusion within tumours and have an obvious impact on leucocyte recruitment by halting rolling and adhesion of leucocytes under the circumstance of malignancy, which was mediated by the downregulation of adhesion molecules in ECs [67,68].

### 3.3. Intraluminal Crawling of Leucocytes

The majority of adherent leucocytes tend to relocate from the initial site of adhesion to the nearest junctional extravasation site in the endothelium before diapedesis, a distinct process termed intraluminal crawling or “locomotion” [69]. This crawling occurs through weak adhesion, using molecules such as macrophage-1 antigen (Mac-1) (or CD11b/CD18) interacting with ICAM-1. Further, blocking of these interactions renders the leucocytes ’blind’ to the endothelial junctions (Table 3) [70]. Actin polymerization serves as a driving force to push leucocytes in the crawling motion [71] while fluid shear changes the kinetics of crawling. Ryschich et al. uncovered, using digital time-lapse intravital microscopy, that most adherent leucocytes (84 ± 13%) crawled actively on the intraluminal site of venules with a 150 μm maximum crawling distance and a 15 min maximum crawling time [72,73].

In tumours, leucocytes exhibit an effective amoeboid movement that is characterised by pseudopod protrusion at the leading edge of the cell, followed by directional locomotion toward a chemotactic source derived from the tumour tissue [74]. Within tumour vessels intravital imaging approaches to visualise the leucocyte-endothelium interactions, Turk and co-workers identified few crawling Ly6G^+^ neutrophils in the venules while CD8^+^ T cells crawled in both collecting and post-capillary venules, indicating different leucocyte subpopulations may crawl along distinct vascular structures or components within the TME (extracellular matrix, collagen, etc.) [75]. Further, it was also revealed that intratumoral leucocytes of both lymphoid and myeloid origin showed an active non-altered intraluminal migration in the mouse hepatocellular cancer model, and the locomotion velocity of leucocytes was also cell number-independent [76]. To better elucidate the potential regulation mechanisms of leucocyte intraluminal crawling in tumour environment during post-irradiation tumour growth, RNA-seq analysis was used and it was shown that enrichment for leucocyte locomotion-associated genes closely correlated with TRP53-regulated, radiation-induced endothelial-to-mesenchymal transition (EndMT), which has been reported to be involved in disruption of endothelial barrier and loss of endothelial adhesion molecules [77,78].

### 3.4. Leucocyte Transendothelial Migration (TEM)

The infiltration of leucocytes across the vascular wall into tissues (namely leucocyte TEM or diapedesis) is an indispensable physiological behaviour that happens during both the adaptive and innate immune response and in the process of immune surveillance and homing [79]. Only activated ECs are capable of triggering leucocyte transmigration since quiescent ECs normally fail to generate the array of vital adhesion molecules and chemokines [80]. The activation of ECs is predominantly through activation of the NF-κB signalling pathway [81,82]. TEM occurs through two mechanistically different routes: the paracellular route and the transcellular route. Paracellular transmigration (moving between ECs) is regarded as the most common type of leucocyte transmigration, which requires transient junctional opening “alongside of” or “beside” adjacent ECs [83,84]. Leucocytes can also transmigrate directly through or across an individual EC cytoplasm using a transcellular route that largely requires the formation of a pore or channel [85]. The recent reemergence in the recognition of the leucocyte transcellular mode is likely due to more detailed understanding of membrane structures which potentially propel transcellular pore formation [86]. Once across the EC barrier, leucocytes have to further traverse a second barrier which is the underlying perivascular basement membrane though they preferentially transmigrate at sites of low matrix protein deposition [87]. Both the paracellular and transcellular migration cascades have similar as well as different regulatory molecules [88], and these are discussed as below (Table 4).

#### 3.4.1. Paracellular Migration

Paracellular transmigration requires the adhesion of the leucocyte to the endothelium, but also needs the clustering of ICAM-1 and VCAM-1 which result in the formation of docking structures or transmigratory cups on the endothelial apical surface membrane [89]. The clustering triggers Src-dependent phosphorylation of cortactin and also actin polymerisation [90]. Importantly, although loosening of the EC junctions is essential for effective paracellular migration [91], this is not associated with vascular leak [92]. VE-cadherin is a central mediator of endothelial barrier integrity [93].VE-cadherin phosphorylation at tyrosine residues 658 and 731 is associated with its internalisation, playing a key role in destabilising EC junctions and thus facilitating paracellular migration of leucocytes [94]. Additionally, vascular endothelial protein tyrosine phosphatase (VE-PTP) and VE-cadherin can form a complex, restricting VE-cadherin at EC junctions. The interaction of leucocytes and activated EC induces transient disassembly of the complex, which enables VE-cadherin to be phosphorylated, thereby promoting TEM [95]. Many other signalling molecules relevant to endothelial junctions influence leucocyte TEM. For example, tight junction proteins including occludin, claudin-5, and claudin-3 preserve the endothelial barrier by sealing the intercellular gaps with membrane and thus hinder the paracellular migration of leucocytes [96]. On the other hand, enhanced myosin light chain (MLC) phosphorylation contributes to increased EC contraction, paracellular gap formation, and boosts leucocyte TEM [97].

As leucocytes transmigrate through the EC barrier, they are encapsulated by the lateral border recycling compartment (LBRC), a recently described peri-junctional network of interconnected tubulovesicular membrane in ECs [98,99]. Targeted trafficking of the LBRC is coordinated with remodelling of EC junctions at sites of TEM [100]. LBRC consists of molecules crucial for leucocyte TEM, such as platelet-endothelial cell adhesion molecule-1 (PECAM-1, CD31), CD99, and junctional adhesion molecule (JAM)-A. Leucocytes interact with these unligated molecules presented by the LBRC membrane on their path across the ECs [88]. Blockade of PECAM or CD99 disrupts the targeted recycling of the LBRC and thus hampered TEM [101]. Further, another key molecule of LBRC, JAM-A, acts as a counter-receptor for LFA-1 that is ideally situated to control transmigration of leucocytes [102].

#### 3.4.2. Transcellular Migration 

Leucocytes are also able to transmigrate via a transcellular mode, whereby the leucocytes are taken up by ECs and are transported in a vesicle-like component from the luminal to the abluminal side [4]. The principal mechanisms of how transcellular transmigration is initiated and what molecules are involved in this process, still remains elusive. Caveolae, fenestrae, and vesiculo-vacuolar organelles (VVOs) are found within ECs and modulate microvascular permeability and transcellular migration [103,104,105]. Interestingly, knockdown of caveolin-1, with short-interfering RNA (siRNA) only repressed the transcellular rather than the paracellular mode of leucocyte migration [106]. In parallel, Marmon et al. reported high levels of caveolin-1 in ECs which preferentially supported the transcellular route whereas its downregulation improved the paracellular mode [107]. Additionally, other influencing elements that might potentially strengthen the transcellular migration of leucocytes include the polygonal shape of the ECs or the levels of β_2_-integrin occupancy, appropriate shear stress, and the existence of a range of key chemokines on the ECs [108,109,110].

In the process of transcellular migration, LBRC membrane surrounds the leucocyte and ICAM-1 expressed on the apical surface of ECs is enriched around the leucocyte adhesion sites [111]. This ensures that the junctions of the EC remain intact [112] and the transcellular migration event is accompanied with augmented expression of ICAM-1 following the stimulation of TNF-α [108]. Interestingly, depolymerisation of microtubules showed no obvious impact on ICAM-1 enrichment, but impeded targeted trafficking of LBRC membrane and transcellular migration of leucocytes [113]. Additionally, in agreement with paracellular migration, the formation of a transmigratory cup made of ICAM-1 clusters and of docking structures as well as the recruitment of PECAM-1, CD99, and JAM-A to leucocytes–ECs interactions via the LBRC can also be the prerequisite for the progression of transcellular migration [86,113]. Taken together, the key discrepancy between the two modes of transmigration lies in the pattern of the vesicles to be attracted to the site of transmigration. In other words, caveolae- and VVO-induced membrane fusion between leucocytes and ECs is deemed to be specific to transcellular migration [114].

#### 3.4.3. Leucocyte Transmigration in Tumour Blood Vessels 

Tumour blood vessels, in contrast to normal blood vessels, present aberrant vascular structures and functions that inhibit leucocyte transmigration [37]. VEGF secreted by tumours diminished TNF-α-mediated leucocyte transmigration through inhibition of leucocyte-attracting chemokines, including C-X-C motif chemokine ligand 10 (CXCL10) and CXCL11, IFN regulatory factor 1 (IRF-1), as well as impairing the phosphorylation level of signal transducer and activator of transcription 1 (p-Stat1). As a consequence, suppression of VEGF signalling pathway by the treatment with sunitinib in tumour-bearing mice led to pronounced upregulation of CXCL10 and CXCL11 in tumour blood vessels, accompanied by a robust increase in the infiltration of leucocytes in tumours [82]. The vasoconstrictive peptide, Endothelin 1 (ET1), can enhance tumour angiogenesis via promoting the levels of VEGF and hypoxia inducible factor-1 (HIF-1) [115,116]. This is associated with reduced leucocyte transmigration via decreased expression of ICAM-1 [117]. In agreement with this, tumour angiogenesis inhibitors promote the transmigration of leucocytes. For instance, Dirkx et al. utilised a panel of angiostatic molecules to decipher that anti-angiogenesis therapy can be a tool to intensify leucocyte–EC interactions and subsequent transmigration into tumours [54]. Intriguingly, Borgstrom and colleagues explored the extent of lymphotoxin (TNF-β) to initiate the transmigration of leucocytes in Lewis lung carcinoma (LLC) model. They uncovered that superfusion of the lymphotoxin-stimulated tumour vessels with LTB4 induced significant leucocyte transmigration, which was prominently prevented in the presence of anti-integrin β_2_ mAb 2E6 [66].

Indeed, neutrophil transmigration was markedly enhanced within the tumour blood vessels, which has been thought to be linked to the increased stiffness of ECs [118]. The tumour neovasculature is also extremely fenestrated due to breakdown of the endothelial junctions by promoting formation of intra-endothelial gaps [119]. Since neutrophils preferentially pursue a paracellular TEM route, they are more prone to transmigrate into tumour parenchyma mainly through the pre-existing gaps at cell borders where three ECs meet [120]. This event was also closely associated with several potential chemotactic factors, among which CXCL8 (or IL-8) has been deemed as one of the most potent neutrophil chemoattractants with respect to tumour vascular biology [121].

## 4. Trafficking of Leucocytes Subpopulation in Tumour Blood Vessels

Leucocytes, or white blood cells (WBC), exert fundamental effects on the human immune system. They can be divided into three main subpopulations: lymphocytes, monocytes, and granulocytes. The overall survival of cancer patients is correlated with type and level of leucocyte subpopulations in the tumour parenchyma, with good survival associated with CD8^+^ T cells, NKs, and DCs, and poor survival associated with neutrophils, Tregs, etc. Thus, efficient admission to these well-defined leucocyte subpopulations presents conspicuous value in research as well as in clinical applications [122,123]. In the tumour setting, selective control of leucocyte trafficking plays a key role in the establishment of effective immune responses against tumour cells [124]. Although targeting the composition and the metabolic state of tumour-associated leucocytes has been proposed as a possibility in altering leucocyte trafficking, emerging evidence has suggested that the regulation of tumour blood vessels may represent a new promising intervention strategy (Table 5) [125]. Therefore, in this section, we will discuss how modulation of tumour blood vessels may influence the trafficking of different leucocyte subpopulations.

**Table 5:** Vasculature-targeting strategies for selectively controlling the trafficking of leucocyte subpopulations.

Leucocyte Subpopulation		Vasculature-Targeting Strategy		Leucocyte Trafficking Phenotype	Reference
T-lymphocyte trafficking		Vascular Normalisation	Deletion of RGS5	Increased CD8^+^ T cell infiltration	[126]
			Anti-VEGFR2 therapy	Increased CD8^+^ T cell infiltration	[18]
			Dual inhibition of VEGF-A and Ang-2	Increased CD8^+^ T cell infiltration	[127]
			Anti-CTLA4 or anti-PD1	Increased CD8^+^ T cell infiltration	[128]
			Increase VE-Cadherin level	Increased CD8^+^ T cell transcellular migration	[20,34]
			Inhibition of FasL	Protect CD8^+^ T cells	[2,129]
		HEVs	Combining anti-VEGFR2 and anti-PD-L1 antibodies (activation of LTβR signalling)	Increased CD8^+^ T cell infiltration	[130]
			LIGHT peptide	Increased CD8^+^ T cell infiltration	[131]
			Augmented levels of various chemokines (CCL19, CCL21, et al.)	Increased CD8^+^ T cell adhesion and transmigration	[132,133]
		Inhibit anergy	Angiogenic inhibitors (PF4, anginex, endostatin, angiostatin)	Increased CD8^+^ T cell adhesion	[58,63]
			Angiostatic factor (16K hPRL et al.)	Increased CD8^+^ T cell adhesion and transmigration	[134]
			DNMT or HDAC inhibitors	Increased CD8^+^ T cell adhesion and transmigration	[135]
Monocyte trafficking	Macrophage	M2-like	Combining anti-PD-L1 and CSF1R	Decreased M2-like macrophage trafficking	[136]
			Inhibition of hedgehog signalling	Decreased M2-like macrophage trafficking	[137]
			CCL2 inhibitor bindarit	Decreased M2-like macrophage trafficking	[138]
		M1-like	Downregulation of PIGF	Increased M1-like macrophage trafficking	[46]
			Activation of STING	Increased M1-like macrophage trafficking	[139]
	DCs	Hypoxia, oxidized low density lipoprotein or TNF-α		Increased DC adhesion and transmigration	[140]
		Deletion of COX		Increased DC infiltration	[141,142]
		Repression of mTORC1		Increased DC infiltration	[143]
		Anti-VEGF therapy		Increased DC infiltration	[144]
Granulocyte trafficking	Neutrophils	Silence of some chemokines (CXCL1, et al.)		Decreased neutrophil infiltration	[145]
		Increase VE-Cadherin expression		Decreased neutrophil adhesion and transmigration	[34,146]
		Dual inhibition of VRGF and Ang2		Decreased neutrophil infiltration	[147]
		RvD1, RvE1 and ATLa		Decreased neutrophil infiltration	[148]
		Suppression of Semaphorin 7A		Decreased neutrophil infiltration	[149,150]
	Eosinophils	Anti-CTLA4 therapy		Increased eosinophil infiltration	[151]
		TNF-α and IFN-γ		Eosinophilic secretion of Th1-type chemokines	[152]
		IL-4		Eosinophilic production of Th2-type chemokines	[152]

### 4.1. T-Lymphocyte Trafficking 

Lymphocytes are regarded as an essential subset of leucocytes that are the mediators of immunity. Lymphocytes consist of three major subpopulations: T cells, B cells, and natural killer (NK) cells [153]. Among these three subsets, T lymphocytes predominantly guide cell-mediated immunity via the secretion of various crucial cytokines and affect the behaviour of other immune-related cells [154]. T lymphocytes play a pivotal role in cancer immunity. In cancer patients with enhanced survival rates there is substantial CD8^+^ T cell infiltrate, and impressive clinical responses to a plethora of immunotherapies that reinvigorate them [155]. Of note, one of the most significant aims of immunotherapy is to convert a so called “cold” tumour, that lack immune effector T lymphocytes, into a “hot” tumour with elevated infiltration of CD8^+^ T lymphocytes [156]. Given that the ultimate purpose of cancer immunotherapy is to accelerate high-avidity tumour-specific T cells to transmigrate across tumour blood vessels and kill malignant tumour cells, strategies that govern trafficking of T lymphocytes via targeting tumour blood vessels are gaining more and more attention. In particular, tumour vascular normalisation has deemed to be an important route to improve immunotherapy as evidenced by enhanced T lymphocyte trafficking [18].

#### 4.1.1. Normalised Tumour Blood Vessels Affect T Lymphocyte Trafficking 

A growing body of evidence has shown that tumour blood vessels set up difficult hurdles for the trafficking of T lymphocytes, in particular effector CD8^+^ T cells [39,144]. Abnormality in tumour vascular structure (tortuous, irregular and dilated blood vessels as well as abnormal pericyte and basement membrane coverage) may initially create a physical barrier to T cell trafficking and infiltration [16,18]. The abnormal tumour vasculature is also able to generate a hypoxic TME, which in turn increases the expression of a wealth of immune checkpoints including PD-L1, CD47, VISTA, and 4-1BB (CD137) [157]. This not only resists the access of T cells to intravasate into tumours even in the presence of CTLA-4 and PD-1 blockade, but also significantly impairs T cell activity [144]. A range of crucial molecules expressed on tumour ECs yield an endothelial barrier that represses T cell arrest. For instance, tumour ECs are capable of boosting the level of Fas ligand (FasL) and thereby selectively kill effector CD8^+^ T cells in the presence of tumour-derived VEGF, prostaglandin E2 and IL-10 stimulation [2,129]. Hence, a decrease of endothelial FasL through VEGF and PGE2 inhibition may be essential for restoring T cell trafficking within TME.

Although normalisation of tumour blood vessels was perceived as a mechanism to limit vessel expansion and decrease the hypoxic TEM, one of the unexpected outcomes is the return to a more controlled regulation of leucocyte trafficking. Normalisation strategies where such effects have been studied are summarised as below.

Pericytes: In tumours, pericytes appear to be absent or loosely attached onto the ECs, which results in the tumour blood vessels becoming more permeable than normal vessels. The high permeability of the tumour blood vessels triggers irregular blood flow, contributing to insufficient T cell trafficking within the tumour parenchyma [158]. An elegant study by Hamzah and co-workers uncovered that tumour-resident pericytes in RGS5 knockout mice exhibited a more normal mature phenotype and the tumour blood vessels were normalised, which contributed to a remarkable decrease in tumour hypoxia and vascular permeability, and increased trafficking of effector CD8^+^ T cells [126]. In addition, Yang et al. revealed that the stimulator of IFN genes (STING) agonist, RR-CDA, conferred elevated pericyte coverage and a remarkable infiltration of CD8^+^ T cells into the TME. In particular, boosted pericyte coverage coincided with the time of peak CD8^+^ T cell infiltration [159].

EC junctions: We have reported that specific upregulation of VE-cadherin by a novel microRNA-based agent, CD5-2, could transform the tumour vasculature to a more normalised phenotype with improved endothelial junctions, and reduced hypoxic TEM. The normalisation resulted in increased CD8^+^ T cells transmigrating into the tumour bed as well as enhanced CD8^+^ T cell activity [34]. Interestingly, the increased transmigration of CD8^+^ T cells into tumours was mainly through the transcellular route, which was mediated via AKT/GSK3β/β-catenin signalling pathway in ECs [20]. This was consistent with a previous study stating that CD8^+^ T cells preferentially pursued transcellular migration when EC junctions became tight [106].

Hypoxia: Hypoxia has been widely held to be the driving force that leads to dysfunctional vascularisation [160]. To this end, alleviation of hypoxia in tumours offers an appropriate microenvironment to achieve vascular normalisation and thereby enhance the trafficking of T cells. For example, Maione and colleagues have shown that reduction of hypoxia by overexpression of semaphorin 3A (Sema3A) contributed to normalised tumour blood vessels, which pave the way for T cell trafficking into tumours [161]. Jayaprakash and colleagues used a hypoxia-reducing drug TH-302 that resulted in a marked influx of T cells into the tumour bed, which was enhanced following the treatment of immune checkpoint inhibitors [162].

Anti-angiogenic therapy: Pro-angiogenic growth factors produced by tumour cells can downregulate the expression of a collection of adhesion molecules (e.g., VCAM-1 and ICAM-1), inhibiting extravasation of T lymphocytes across the tumour endothelium [2,157]. Disruption of tumour vascular structure with anti-angiogenic agents led to the restoration of homogenous blood flow, improving the trafficking of effector CD8^+^ T cells [38]. Low-dose of anti-VEGFR2 antibody DC101 gave rise to a more homogeneous distribution of functional tumour blood vessels and further promoted the infiltration of CD8^+^ T cells into tumours [18]. Moreover, immune checkpoint blockade with either cytotoxic T lymphocyte–associated protein 4 (CTLA4) or PD1 antibody has been shown to elicit tumour vessel perfusion, conferring a significant increase in CD8^+^ T cell infiltration into tumours [128]. Additionally, dual inhibition of VEGF-A and angiopoietin-2 (Ang-2) induced vascular normalisation and facilitated the transmigration of activated CD8^+^ cytotoxic T cells into tumours, improving outcomes when in combination with anti-PD-1 in various mouse tumour models [127].

Other strategies: Recently, a variety of new strategies to bridge tumour vascular normalization and CD8^+^ T cell infiltration have been gaining attention. For instance, bone morphogenetic protein 9 (BMP9) was reported to promote the normalisation of tumour blood vessels via activating activin receptor-like kinase 1 (Alk1) signalling, yielding increased influx of T cells [163]. Although hypoxia-inducible factor 2 alpha (HIF-2α) is known to be involved in the response to hypoxia, endothelial deletion of HIF-2α leads to disrupted tumour vascular function and stabilisation of HIF-2α frequently exists in tumour vascular normalization [164,165]. Indeed, superoxide dismutase 3 (SOD3) was shown to normalise the blood vessels in tumours and enhance transendothelial migration of effector T cells into tumours through promoting the stabilisation of HIF-2α [166]. Further, ECs tend to make use of fatty acid oxidation for nucleotide synthesis. As such, pharmacological suppression of fatty acid oxidation regulators could normalise tumour vasculature, which paves the way for CD8^+^ T cell infiltration into tumours [29].

#### 4.1.2. High Endothelial Venules (HEVs) in Tumours Control T Lymphocyte Trafficking 

HEVs are anatomically diverse post-capillary venules that emerge as main portals of entry for lymphocytes into lymph nodes and other secondary lymphoid organs [167]. The endothelium expresses a specific group of cell surface mucin-like glycoproteins, named peripheral node addressins (PNAds) with the HEV-specific antibody MECA-79 recognising the specific addressin, MAdCAM1 [168]. HEV ECs are held together by important adherens junction molecules especially VE-cadherin but lack tight junctions and vascular specific claudin-5, resulting in the unique property of HEVs that maintain blood vessel integrity [167].

The density of HEVs is tightly associated with the number of tumour-infiltrating CD3^+^ and CD8^+^ cytotoxic T cells [169]. High densities of tumour-associated HEVs independently contribute to a lower risk of relapse and are significantly related to longer metastasis-free and overall survival rates in a cohort of invasive breast cancer patients [170]. Allen et al. showed that combining anti-VEGFR2 and anti-PD-L1 antibodies induced HEVs in breast cancer and pancreatic cancer models through activation of lymphotoxin β receptor (LTβR) signalling [130]. Further, Johansson and colleagues demonstrated that vascular normalization therapy in combination with a vascular targeting peptide coupled to LIGHT (also called TNF superfamily member 14; TNFSF14) acts as a ligand for the LTβR, and enhanced the formation of HEVs and tertiary lymphoid structures in pancreatic neuroendocrine tumours [131]. Notably, cells that compose of LIGHT-mediated HEV structures express both MAdCAM1 and PNAd, which can bind L-selectin on T lymphocytes and facilitate T effector cell transmigration into tumours [131,171].

In HEV^high^ tumours compared with HEV^low^ tumours, gene profile of HEVs has shown upregulation of genes encoding lymphoid chemokines including chemokine (C-C motif) ligand (CCL) 19 (CCL19), CCL21, and CXCL13 as well as T-cell homing receptors (CCR7 and LSEL) [170]. Genetic deficiency in the expression of both CCL19 and CCL21 in the HEVs of mice displayed strikingly impaired T lymphocyte adhesion to HEVs and T cell transmigration [132,133]. Additionally, some other chemokines controlling T lymphocyte homing to peripheral tissues, such as CCL5, CXCL9, CXCL10, and CXCL11, were also highly expressed in tumours containing large numbers of tumour HEVs [172]. Surprisingly, CD11c^+^ DCs were shown to be required for the maintenance of HEVs in an LTα_1_β_2_-dependent manner [173]. In contrast to DCs, Tregs are negative mediators in the formation of tumour HEVs [169]. Thus, modulation of tumour blood vessels to enhance the formation of HEVs with high density is proposed as an effective route to increase T cell trafficking.

#### 4.1.3. Endothelial Cell Anergy Has Impact on T Lymphocyte Trafficking 

Angiogenesis is able to trigger EC anergy, a failure to respond to a large amount of proinflammatory cytokine stimulation such as TNF-α, IL-1, and IFN-γ. This anergy at least partially results from continuous stimulation by angiogenic factors such as VEGF and FGF, which inhibit TNF-α-mediated upregulation of VCAM-1, ICAM-1, and chemokines [54,174,175]. The anergy contributes to decreased leucocyte-vessel wall interactions and thereby diminished inflammatory infiltration [54]. Suppression of tumour angiogenesis, through platelet factor-4 (PF4), endostatin, and angiostatin, and the chemotherapeutic agent paclitaxel could reverse tumour anergy resulting in strengthened lymphocyte–EC associations by increased expression of endothelial adhesion molecules in tumour blood vessels [58,63]. Another novel potent angiostatic factor, 16-kDa N-terminal fragment of human prolactin (16K hPRL), overcame endothelial anergy resulting in high numbers of infiltrated T cells [134]. Further, Hellebrekers et al. demonstrated that epigenetic regulation plays an essential role in curbing tumour EC anergy. They showed that treatment with either DNA methyltransferase (DNMT) or histone deacetylase (HDAC) inhibitors could increase ICAM-1 expression on tumour ECs and thus potentiate leucocyte infiltration in two different mouse tumour models [135]. Collectively, antagonizing tumour endothelial anergy offers an effective pathway to increase T cell trafficking.

### 4.2. Targeting Tumour Blood Vessels in Governing Monocyte Trafficking 

Monocytes are heterogeneous circulating white blood cells that play a fundamental role in tissue homeostasis, protective immunity, and both promotion and resolution of inflammation [176,177]. Similar to other leucocyte subsets, recruitment of monocytes to the site of disease relies on their trafficking across the blood vessel wall although there are tissue resident macrophages [178]. They utilise a similar process as other cells, but with selective chemokines, such as monocyte chemotactic protein (MCP)-1 (CCL2) which facilitates monocytes to transmigrate into inflammatory sites [179]. Integrin α4β1 augments monocyte trafficking and subsequent neovascularisation and specific α4β1 antagonists block the adhesion of monocytes to endothelium as well as their extravasation into tumour tissue [180]. Additionally, transendothelial migration of adherent monocytes needs remodelling of the EC junctions [179]. As for lymphocytes, VE-cadherin is remodeled to allow monocytes to transmigrate [181]. The JAM family that belongs to tight junctions also participates in the process of monocyte transendothelial migration [182]. Given the fact that circulating monocyte can differentiate into either macrophages or DCs, we will discuss the trafficking of these two subsets in tumour blood vessels.

#### 4.2.1. Macrophage Trafficking 

Tumour-associated macrophages (TAMs) predominantly derive from circulating monocytes that stem from the bone marrow [183]. TAMs are capable of increasing angiogenesis, suppressing the anti-tumour immunity especially T-cell-induced cytotoxicity, and producing cytokines that participate in remodelling of extracellular matrix (ECM), therefore promoting tumour cell motility and intravasation [184,185,186]. However, TAMs are also associated with improved anti-tumour immune responses [187,188]. In fact, M2-like TAMs give rise to immunosuppression and production of aberrant tumour blood vessels resulting in tumour progression, while M1-like TAMs are able to induce immunity and normalise disorganised tumour microvascular network that sensitise tumour cells to chemo- and radiotherapy and further cause tumour regression. Hence, the phenotype of TAMs in the TME exert significant effects on tumour vascular abnormalisation/normalisation [46,189]. Macrophages are believed to play a role in extracellular matrix composition either via secretion of degradative enzymes or via regulating extracellular matrix cytokines, thus affecting angiogenesis in tumours [190]. More importantly, Harney and co-workers elucidated that VEGF-A signalling from TIE2^hi^ macrophages led to disruption or loss of endothelial junctions, transient vascular leak, and tumour cell intravasation, providing insight into the mechanism of tumour distant metastasis [191].

TAM trafficking into tumours is dependent on CCL2 expression. The CCL2 inhibitor, bindarit inhibited M2-like macrophage trafficking and reduced human melanoma xenografts [138]. CCL2 antibodies in combination with chemotherapeutic drug docetaxel also decreased tumour burden in prostate cancer [192]. Further, anti-PD-L1 in combination with colony stimulating factor-1 receptor (CSF1R) diminished TAM trafficking but enhanced CD8^+^ T cell infiltration in various HCC mouse models [136]. Indeed, one of the major functions for TAMs is their regulation of T cell infiltration. Depletion of TAMs heightened T cell transmigration and infiltration into tumour parenchyma and further promoted the effectiveness of anti–PD-1 immunotherapy [48]. Hedgehog signalling was involved to inhibit CD8^+^ T cell trafficking into the TME via the repression of CXCL9 and CXCL10 expression [137]. Zhu and colleagues illustrated that the expression of osteopontin (OPN) was positively associated with TAM trafficking in tumour tissues obtained from patients with hepatocellular carcinoma (HCC) [136].

There are a number of approaches being tested for conversion of a M2-rich into an M1-rich TAM microenvironment. For instance, Zoledronic acid, a potent drug for suppression of spontaneous tumour growth, exerted striking anti-angiogenic effect in part through repolarisation of pro-angiogenic M2-like TAMs to suppressive M1-like TAMs [193]. Moreover, histidine-rich glycoprotein was able to repolarise M2-like TAMs to M1-like TAMs with elevated tumour immunity and mediated vascular normalisation via downregulation of placental growth factor (PlGF) [46]. Additionally, Downey et al. demonstrated that a vascular disrupting agent 5,6-dimethylxanthenone-4-acetic acid (DMXAA) transformed M2-like TAMs towards the M1-like phenotype through the activation of STING in mouse tumour models [139]. All of these results suggest that targeting tumour blood vessels has an impact on macrophage trafficking. On the other hand, repolarisation of TAMs from M2-like toward M1-like is an efficient route for tumour vascular normalisation.

#### 4.2.2. DC Trafficking 

DCs act as key orchestrators of the immune response. Monocyte-derived DCs (mo-DCs), also referred to as inflammatory DCs (inf-DCs), can be recruited into tissues and appear to be the most abundant DC population in the process of inflammation [194,195]. Their classic phenotype in mice is characterised by the expression of various macrophage markers such as F4/80, Ly6C, CD64, and FcεR1 [196]. Of note, the robust ability of DCs to trigger and modulate adaptive immune responses supports the successful generation of T-cell-mediated anti-tumour immune response [197]. In particular, the achievement of DCs-based immunotherapy in initiating cellular immune response against tumours depends on delivery and trafficking of the DCs across the endothelium to T-cell-rich areas within tumours [198].

Modulation of DC trafficking has been achieved through a number of different routes. These include (1) activation of the endothelium by exposure to hypoxia, oxidized low density lipoprotein or TNF-α [140], depletion of cyclooxygenase (COX) enzymes in ECs to inhibit the levels of PGE2 and boost the trafficking of DCs within the TME [141,142], and application of sarcosine (N-methylglycine) to enhance the expression of CXC chemokine family including CXCL1 and CXCL3 which promoted the trafficking of DCs and boosted the effectiveness of anti-tumour dendritic cell vaccines [199].

(2) Targeting the extracellular matrix around the tumour blood vessels, which is high in collagen, also can modulate DC infiltration. A fusion protein (CBD-CCL4) of chemokine CCL4 and the collagen-binding domain (CBD) of von Willebrand factor (VWF, specifically the A3 domain) was able to recruit CD103^+^ DCs into the TME and promote the anti-tumour immune response [200]. Furthermore, Wang et al. elucidated that selective repression of mTORC1 in ECs with an mTORC1 inhibitor, RAD001, contributed to tumour vascular normalisation and further enhanced immune response with elevated CD103^+^ DC infiltration [143].

(3) Targeting tumour vasculature associated signalling pathways, including blockade of VEGF-A and ANG2 reinforced the antigen-presenting ability of DCs, and the combined therapy potentiated DCs to present an activated phenotype characterised by increased expression of MHC-II and CD86 [201]. VEGF binding to VEGFR1 on the endothelium can inhibit the maturation of DCs from immature precursors and thus disrupt T-cell priming against tumours [202]. Hence, anti-VEGF therapy has been reported to ameliorate the VEGF-mediated immunosuppression on DCs via both diminishing immature progenitors and accelerating the maturation of DCs [144].

### 4.3. Granulocyte Trafficking 

Granulocytes or polymorphonucleocytes are characterised by the presence of granules and include neutrophils, eosinophils, and basophils [203], and are critical for infection control. Several critical adhesion molecules including P-selectin and ICAM-1 actively participate in the binding of granulocytes onto ECs [204]. In addition, Koskinen et al. reported that vascular adhesion protein-1 (VAP-1) as an EC-surface enzyme (amine oxidase) was involved in the rolling and transmigration of the granulocytes under flow. Therefore, blockage of VAP-1 resulted in at least 50% suppression of granulocyte transmigration, with the other adhesion molecules expressed constitutively or stimulated by TNF-α on the ECs staying intact [205]. Another chemokine named granulocyte chemotactic protein-2 (GCP-2) or CXCL6 was shown to play an important role in promoting granulocyte recruitment [206]. Of note, granulocytes obtained suppressive or granulocytic-myeloid-derived suppressor cell (G-MDSC) ability in particular under the circumstance of tumour development [207]. Here, we mainly review the trafficking of two granulocyte subsets, neutrophils and eosinophils. Neutrophils in particular are linked to a bad prognosis for cancer patients [208] since they can inhibit T-cell immunity [209]. Hence the aim in immunotherapy would be selective inhibition of neutrophil recruitment, but enhanced T cell recruitment.

#### 4.3.1. Neutrophil Trafficking

Neutrophils are the most enriched cell population in the immune system [210] and have been one of the major cell types used to dissect leucocyte transendothelial cell migration. Deep insight into mechanisms of neutrophil trafficking into different organs including tumours has been gained. It is now appreciated that the mechanisms of neutrophil trafficking in the same organ may vary with different inflammatory stimuli [211]. Various adhesion receptors (CD44, PSGL-1, Syndecan-1, CXCR1, et al.) on neutrophils spatially and temporally interact with unique ligands expressed on endothelium (e.g., E-selectin, P-selectin, ICAM-1, CXCL8) during the trafficking of neutrophils [212]. Neutrophil ‘slings’ (membrane tethering structures) allow interaction with the vessel wall at high shear stress [213]. Subsequent, firm attachment is mediated by LFA-1 and Mac-1, members of the β_2_ integrin family that are predominantly expressed on neutrophils, binding to ICAM-1 expressed on ECs [214], and finally transmigration occurs predominantly through endothelial junctions (paracellular fashion, ~90%) rather than directly via the bodies of ECs (transcellular fashion, ~10%) [215]. High neutrophil content of tumours is a poor prognosis [208] and CD177-positive neutrophil infiltration seemed to act as a predictor of adverse clinical response to anti-VEGF therapy in cancer patients [147].

The transmigration of neutrophils into the tumour parenchyma can occur by enhanced expression of adhesion molecules and chemokines which include CXCL1, CXCL8 and CCL15 [216,217,218]. Indeed, silencing of CXCL1 levels in tumour cells dramatically retarded tumour growth by preventing the infiltration of neutrophils from peripheral blood into tumour sites [145]. The potential to target neutrophils in tumours, is complicated by their dual role. On one hand, tumour-associated neutrophils (TANs) act as part of the tumour-intensifying inflammation by escalating angiogenesis, extracellular matrix remodelling, and immunosuppression. In fact, TANs in tumour-bearing mice produced CCL17 to provoke the infiltration of Tregs into tumour parenchyma, thus potentiating tumour progression and emerging as ideal targets for anti-tumour therapy [219,220]. On the other hand, neutrophils may also be involved in the anti-tumour response by directly destroying malignant tumour cells and by their involvement in cellular networks that incite anti-tumour resistance [221].

Normalising tumour vasculature via improving the endothelial junctions (mainly through increasing VE-cadherin expression) resulted in decreased neutrophil infiltration into tumour parenchyma [34,146]. Further, Schiffmann and colleagues found that dual inhibition of VEGF and Ang2 with nanobody BI-880 could overcome neutrophil associated resistance of anti-VEGF treatment [147]. Additionally, Jin et al. elucidated that resolvin D1 (RvD1), resolvin E1 (RvE1), and a stable analogue of aspirin-triggered lipoxin A4 (ATLa) profoundly impaired the levels of angiogenic growth factors and their receptors and thus the infiltration of neutrophils [148]. Intriguingly, Morote-Garcia and colleagues identified that endothelial Semaphorin 7A, a potent pro-angiogenic mediator, worked to reinforce inflammatory damage through enhancing neutrophil trafficking [149]. Hence, suppression of Semaphorin 7A led to impaired tumour progression in a murine model of advanced breast cancer [150]. To this end, inhibition of tumour angiogenesis or achievement of vascular normalisation abolished the trafficking of neutrophils in tumours, leading to repression of tumour growth and metastasis.

#### 4.3.2. Eosinophil Trafficking

Eosinophil accumulation in the peripheral blood and tissues is a typic hallmark of multiple common diseases such as atopic disorders, parasitic infections, and cancer [222]. Similar to neutrophils, eosinophils in tumours display both pro- and anti-tumourigenic roles. Eosinophils can produce pro-angiogenic and matrix-remodelling soluble contributors (e.g., EGF, TGF-β1, MMP-2, MMP-9) that exacerbate tumour growth. Additionally, eosinophils are able to secrete a large number of mediators, including specific granule proteins that can eradicate tumour cells [223]. In some solid tumours, eosinophils are more prone to have a T cell-independent anti-tumour immune response [224]. However, it has also been shown that activated eosinophils enhanced tumour rejection in the presence of tumour-specific CD8^+^ T cells, through action to alter the TME with increased IFN-γ and TNF and to induce tumour vascular normalisation [225]. Further, anti-CTLA4 therapy-mediated tumour vascular normalisation was accompanied by a striking escalated infiltration of eosinophils into tumours, which was dependent on T lymphocytes and IFN-γ production [151]. Notably, different cytokines may play different roles in modulating the trafficking of eosinophils. For instance, TNF-α and IFN-γ tend to boost eosinophilic secretion of pro-inflammatory Th1-type chemokines, including CXCL9 and CXCL10. Nevertheless, IL-4 appeared to heighten eosinophilic production of Th2-type chemokines that create a more immunosuppressive TME [152]. However, whether the diverse nature of eosinophils in tumour progression is associated with abnormal tumour endothelium requires further investigation.

## 5. Conclusions

The goal of immunotherapy is to harness the immune response to deliver tumour killing potential. However, the immune suppressive nature of the tumour microenvironment hinders the infiltration of leucocytes into tumour parenchyma. Thus, strategies for converting “cold tumours” into “hot tumours” as evidenced by increased and appropriate leucocyte infiltration into tumour parenchyma, have gained attention. Since blood vessels, and in particular the endothelium, are central players in controlling the delivery of leucocytes into tissue, it has become a new target for anti-cancer therapies. Indeed, anti-angiogenic treatment can promote leucocyte trafficking and reduce the number of newly formed blood vessels. Further, vascular normalisation as a novel vasculature-targeting therapy facilitates the trafficking of leucocytes via improving tumour vascular structure and function. Of particular note now are strategies that will enhance selective immune subsets and will aid their activation while inhibiting access of detrimental subsets into the tumour to improve cancer immunotherapy and bring great benefit for cancer patients.

## Figures and Tables

**Table 1:** Mechanisms involved in leucocyte capture.

Mechanisms Involved in Leucocyte Capture			
Primary Capture		Secondary Capture	
Molecules on ECs	Molecules on Leucocytes	Molecules on ECs	Molecules on Leucocytes
P-selectin	PSGL-1	N/A	L-selectin, PSGL-1
E-selectin	PSGL-1		

**Table 2:** Mechanisms involved in leucocyte rolling and adhesion.

Mechanisms Involved in Leucocyte Rolling and Adhesion	
Molecules on ECs	Molecules on Leucocytes
P-selectin	PSGL-1
E-selectin	PSGL-1
ICAM-1	LFA-1, Mac-1
VCAM-1	VLA-4, α4β1-integrin
MAdCAM-1	α4β7-integrin

**Table 3:** Mechanisms involved in intraluminal crawling of leucocytes.

Mechanisms Involved in Intraluminal Crawling of Leucocytes	
Molecules on ECs	Molecules on Leucocytes
Chemokines	Chemokine receptors
ICAM-1	LFA-1, Mac-1
VCAM-1	VLA-4, α4β1-integrin

**Table 4:** Mechanisms involved in leucocyte TEM.

Mechanisms Involved in Leucocyte TEM			
Paracellular Migration		Transcellular Migration	
Molecules on ECs	Molecules on Leucocytes	Molecules on ECs	Molecules on Leucocytes
VE-cadherin	N/A	PECAM-1	PECAM-1
PECAM-1	PECAM-1	JAM-A	LFA-1
JAM-A	LFA-1	CD99	CD99
CD99	CD99	ICAM-1	LFA-1, Mac-1
ICAM-1	LFA-1, Mac-1	Caveolin-1	N/A
Chemokines	Chemokine receptors	Chemokines	Chemokine receptors
